# A bite of dark chocolate? Black humour in mental health services

**DOI:** 10.1177/10398562231222815

**Published:** 2023-12-21

**Authors:** Aram Kim, Rosie Luo, Lillian Ng

**Affiliations:** 1415 The University of Auckland, Auckland, New Zealand and 637385 Health New Zealand Te Whatu Ora Waitemata, Auckland, New Zealand; 1415 The University of Auckland, Auckland, New Zealand; 1415 The University of Auckland, Auckland, New Zealand and 637385 Health New Zealand Te Whatu Ora Counties Manukau, Auckland, New Zealand

**Keywords:** black humour, psychiatry, mental health services

## Abstract

**Objective:**

Black humour permits expression of what may otherwise be unspeakable and is observed and used by staff working in mental health services. The aim of this study was to identify how humour, particularly black humour, was perceived by different health professionals in psychiatric practice.

**Methods:**

Participants were invited to complete a survey. Data was collated and statistically analysed by a biostatistician. Chi square and univariate tests were performed to identify associations between categories.

**Results:**

The sub-question relating to the benefits of black humour was analysed. Main findings were that the majority of staff perceived black humour to be beneficial in psychiatric practice (*n* = 564 of 710 total; 79.4%), particularly if they used general and black humour with patients, families and colleagues. Those who observed black humour being used collegially about patients and families were more likely to find black humour beneficial; and even those uncomfortable with black humour being used by colleagues were more likely to see the benefits of black humour.

**Conclusion:**

Black humour was perceived to be beneficial in mental health settings when used mindfully, sensitively and in context. Further study with patients and relatives may shed light on how widely the perception of benefit is shared.

Humour, the ‘quality of being amusing or comic’,^1^ contributes to physical^2^ and psychological wellbeing^3^ with favourable effects on cardiovascular and immune systems.^4^ Humour enhances positive emotions, moderates stress and improves interpersonal relationships.^3^ The propensity to laugh^5^ is a mature and elegant form of defence,^6^ enabling the ego to triumph over adversity.^7^

Health professionals use humour to build rapport or relieve tension^4^ amidst a crisis.^8^ Humour can be a coping strategy^9^ in response to challenging clinical work^5^ which is fraught with intensity and potentially perilous. Black humour is a specific form of humour that juxtaposes tragic with comic elements,^1^ underscoring nonsensical or futile elements of life. This juxtaposition of two incongruous frames of reference is essentially a shift in cognitive perspective, which may allow distance from the immediate threat and reduce feelings of anxiety and helplessness.^4^ Black humour may offend when human suffering is perceived as being made light of rather than an intent to make sense of what is absurd or ironic. Farce or low comedy portrays individuals as helpless victims of fate and character who walk a fine line between being boorish and funny.^10^

In this context of being privy to suffering, black humour may permit us to articulate what is otherwise unspeakable. Yet attempts to lighten the tone in a bleak situation may be deemed inappropriate and cause discomfort. Humour is negatively perceived when there are malevolent undertones, when someone is seen to be subjectively devalued, inferior or demeaned. There is a great deal of variation in individuals’ responses to similar life events,^4^ and there is potential for humour to unsettle a person’s emotions in a negative way. Because humour is subjective, mental health professionals need to be conscious of perils that accompany inappropriate use of humour with those in mental distress.^11^ Clinicians working within a multidisciplinary team observe humorous exchanges between members of staff, patients and family. In this study, we aimed to identify how humour was perceived by different health professionals.^12^ In this article, we focused on the analysis of black humour in the psychiatric context.

## Methods

A survey was designed to identify comfort and perceived benefit in observing or using black humour, and humour more generally and associations with discipline, ethnicity and length of work experience. The study was approved by the New Zealand Health and Disability Ethics Committee.

The original survey instrument contained 13 questions with four stems: demographic information, colleagues’ use of humour, use of humour with patients and the perception of dark humour as harmful. Humour was defined as a message whose ingenuity or verbal skill or incongruity has the power to evoke laughter or has the quality of being amusing. Black humour was defined as a form of humour that regards human suffering as absurd rather than pitiable or that considers human existence as ironic and pointless but somehow comic. Participants were asked to briefly describe memorable positive and negative experiences of humour in the workplace. The initial survey questions were piloted and then revised.

Inclusion criteria were participants employed in mental health and addiction services in a metropolitan area. The study was advertised by email from coordinators of mental health services attached with a participant information sheet and a link to the survey. Participants gave written consent to take part in the study prior to completing the survey.

Completed responses were stored online in the SurveyMonkey cloud and downloaded once the survey link had expired. For this study, we analysed the subset of black humour relating to the question, ‘Do you think black humour is beneficial?’ The data was collated in pivot tables using an Excel spread sheet. A biostatistician assisted with statistical analysis, using SAS software. Chi square and univariate tests were performed to identify associations between categories. In the data cleaning phase, it became apparent that the Likert scale required simplification: as such ‘never/rarely and sometimes/always’ were condensed into no and yes, respectively.

## Results

The question in the survey, ‘Do you think black humour was beneficial?’ was completed by 710 participants from medical, nursing, allied health and other disciplines which included pharmacists, alcohol and drug clinicians, cultural support workers and administrators (Table 1). There were variations in responses about the perceived benefits of black humour according to ethnicity: European (82%), Asian (81%), Māori and Pasifika (66%) and other ethnicities (81%). Participants who spoke English as second language were less likely to find black humour beneficial (68% vs 81%). Our main findings were (1) 79% of staff found black humour to be beneficial in psychiatric practice; (2) participants who used humour more generally with patients, families and colleagues were more likely to find black humour beneficial (with patients and families 81% vs 60%; with colleagues 87% vs 60%); (3) participants who used black humour with patients, families and colleagues were more likely to find black humour beneficial (with patients and families 96% vs 74% and 90% vs 63%); (4) participants who observed black humour being used in a collegial way with other colleagues about patients and families were more likely to find black humour beneficial (90% vs 63%) and (5) participants who were uncomfortable with black humour being used by colleagues were less likely to find black humour beneficial (72% vs 82%). Freeflow text responses (*n* = 345) illustrated how black humour defused emotions and humour in general was seen to be a way of coping with difficult situations (Table 2). Negative examples of humour were also reported.

**Table 1.** Statistical analysis: Use and perceptions of black humour

Do you think black humour is beneficial?				
	Total	Black humour is not beneficial	Black humour is beneficial	*p*-value
Designation				
Allied health	204	48 (23.5%)	156 (76.5%)	.0003
Doctor	105	20 (19.0%)	85 (81.0%)	
Nurse	258	34 (13.2%)	224 (86.8%)	
Other	143	44 (30.8%)	99 (69.2%)	
Total	710	146 (20.6%)	564 (79.4%)	
Gender				
Female	499	100 (20.0%)	399 (80.0%)	.2972
Male	211	46 (21.8%)	165 (78.2%)	
Age categories				
21–35	131	27 (20.6%)	104 (79.4%)	.0072
36–40	95	9 (9.5%)	86 (90.5%)	
41–45	101	17 (16.8%)	84 (83.2%)	
46–50	100	26 (26.0%)	74 (74.0%)	
51–55	134	24 (17.9%)	110 (82.1%)	
56–60	82	20 (24.4%)	62 (75.6%)	
60+	67	23 (34.3%)	44 (66.7%)	
Ethnicity				
Asian	42	8 (19.0%)	34 (81.0%)	<.0087
European	527	96 (18.2%)	431 (81.8%)	
Māori and Pasifika	100	34 (34.0%)	66 (66.0%)	
Other	41	8 (19.5%)	33 (80.5%)	
English as a first language				
No	99	32 (32.3%)	67 (67.7%)	.0029
Yes	611	114 (18.7%)	497 (81.3%)	
How long have you worked in mental health?				
Mean (standard deviation)		14.1 (9.7)	14.5 (10.4)	.6246
Sub-speciality				
CADS/dual diagnosis	79	17 (21.5%)	62 (78.5%)	.5670
Child and adolescent	60	9 (15.0%)	51 (85.0%)	
Cultural	20	6 (30.0%)	14 (70.0%)	
Forensic	79	14 (17.7%)	65 (82.3%)	
General adult	303	63 (20.8%)	240 (79.2%)	
Consult-liaison	21	2 (9.5%)	19 (90.5%)	
Old age	66	14 (21.2%)	52 (78.8%)	
Other	82	21 (25.6%)	61 (74.4%)	
Do you use general humour with patients and families?				
No	58	23 (39.7%)	35 (60.3%)	<.001
Yes	652	123 (18.9%)	529 (81.1%)	
Do you use general humour with colleagues about patients and families?				
No	196	79 (40.3%)	117 (59.7%)	<.001
Yes	514	67 (13.0%)	447 (87.0%)	
Do you use black humour with patients and families?				
No	528	138 (26.1%)	390 (73.9%)	<.001
Yes	182	8 (4.4%)	174 (95.6%)	
Do you use black humour with colleagues about general matters?				
No	159	92 (57.9%)	67 (42.1%)	<.001
Yes	551	54 (9.8%)	497 (90.2%)	
Do your colleagues use black humour with patients and families?				
No	461	119 (25.8%)	342 (74.2%)	<.001
Yes	249	27 (10.8%)	222 (89.2%)	
Do your colleagues use black humour with other colleagues about patients and families?				
No	274	101 (36.9%)	173 (63.1%)	<.001
Yes	436	45 (10.3%)	391 (89.7%)	
Are you uncomfortable with your colleagues’ use of black humour?				
No	537	98 (18.2%)	439 (81.8%)	.0087
Yes	173	48 (27.7%)	125 (72.3%)	

Note: No/Yes is in response to the question directly above.

**Table 2.** Survey free text responses: Perceived benefits of black humour

**Black humour as beneficial in context**
Often use black humour with work colleagues, never with patients or families. It always lightens the situation and bring us all to laugh. I never use it to denigrate anyone.
Black humour was used to make jokes that made everyone laugh and reduced tension
Use black and general humour to defuse situations usually with positive results
Humour is tricky – if with families then my rule is that it has to be initiated by the family. It is interpreted differently by different cultures and also children and adolescents have a different sense of humour and can easily feel belittled or humiliated by humour used by adults. Black humour is even more tricky – if used by teams and I think it is a valid coping mechanism. I think we need also to put into words the awfulness at the same time.
Very occasional use of black humour provides relief and allow us to cope with truly horrible situations.
Many instances where humour has defused a tense situation that was going to become violent.
The use of humour in verbal de-escalation – it provides a human element that patients respond to.
Humour is a very powerful and useful but must be used with the greatest respect.
It gets you through those times you’ve just had enough of it all. Generally only come a cropper in regards to black humour backfiring when it is interpreted for more than what it is (ventilation or coping strategy) when we have colleagues who don’t have the experience of where the humour is coming from so tend to take what is said as insensitive rather than purely expressive ventilation of difficult situations such as suicide and other extremes.
**Concerns about use of black humour**
Staff members in team meeting may make black humour comments about a difficult family and it feels unclear to me whether or not they are using humour to describe their emotions, be judgmental towards the family or relieve their distressing emotions.
Black humour has its place and is okay when used in a very nonspecific way. Can be derogatory and damaging to the environment of recovery, perpetuates stigma and discrimination of mental illness.
Black humour about my religious choices, ethnicity, cultural preferences, family matters is mostly very hurtful and should be avoided.
I’ve frequently felt uncomfortable with the use of both black and general humour in discussions. It was difficult to challenge this as it was an embedded part of culture.
I think of [black humour] as being judgmental and unhelpful as it increases the intensity of emotion or makes light of distress in a way that is not light but disrespectful.
The use of black humour is not really that funny. It is disrespectful at times in what can be a difficult job. I can’t recall any positive experiences.
The use of black humour is not really that funny it is disrespectful at times it takes to each of what can be a difficult job I can’t recall any positive experiences.

## Discussion

Our findings demonstrate that black humour is widely used in clinical psychiatry as a mechanism to release tension^4^ in incongruent situations,^10^ to lighten the tone of a crisis^8^ and to cope with the burden of work.^9^ Many participants were comfortable with black humour. Nurses were more inclined to see black humour as beneficial, which may reflect their disposition to using humour to cope with high intensity and stress.^13^ Participants less likely use black humour may be wary of misinterpretation. If participants did not use humour more generally with colleagues about patients and families, then they were more likely to be uncomfortable observing humour being used by colleagues. This discomfort may be linked to conventional understanding about clinician roles and the therapeutic alliance.^11^

The majority of participants perceived black humour as beneficial in psychiatric practice. With patients and families, familiarity with context helps gauge the appropriateness of humour, particularly black humour. Staff who refrain from using humour with colleagues about patients and families may believe that laughter can be misconstrued, undermining a therapeutic relationship, a service user’s dignity or inadvertently minimise their suffering.

It may be that participants who use general humour with patients and families are comfortable with black humour and attuned to its use and its impact. Those who use black humour with patients and families are perhaps more at ease with humour. Pasifika and those participants who were older or with English as a second language were less likely to identify black humour as beneficial which may reflect cultural nuances of language and experiences of conscious and unconscious bias or racism.

Humour can be considered adaptive,^14^ enabling us to live and work creatively and as a tonic to cope with witnessing suffering.^15^ Health professionals can role model and shape attitudes in using humour.^16^ Survey comments show that staff working in mental health services value humour, particularly black humour, when used sensitively and in context. Staff reported that humour helped them ‘stay sane’, express frustration, cope with stress and eased difficult situations.^17^ Discerning health professionals will be aware that intent to be humorous will not always be perceived as such.^18^ Integrating humour with skill and sensitivity may enhance clinical encounters,^19^ even when people are seriously mentally ill^11^ and strengthen connections within a multidisciplinary team.^12^ When humour is sarcastic, used at the patient’s expense and has sexist or racist connotations,^20^ it is detrimental in eroding relationships and contributes to lowered morale, resentment and vengeful counteraction.^21^ Therefore, mindful use of black humour in the psychiatric context is paramount. Black humour is a communication that may signal unease when the work is most challenging amidst conflicting team dynamics. It may also reflect team members’ experience of paradox and ambiguity in organisations,^12^ lift spirits in the grimmest of times and bring a brighter spark to the darkest of times and spaces.^22^

### Strengths and limitations

The main strength of this study is the high number of responses received from a diverse range of disciplines and ethnicities. Limitations include the potential for the survey to have been completed more than once by individual participants, the limits of the survey tool in the way questions were posed to participants and the use of broad Likert scales. Another limitation is the difficulty of defining humour, which can be considered highly subjective. An alternative approach may be to provide a concrete hypothetical example of black humour and ask survey participants to respond to this specific example. The large volume of data required cleaning and categories were condensed by a biostatistician in the analysis phase. We report findings from a subsection of the survey specific to black humour. The text responses give a glimpse of how black humour is observed in psychiatric practice. There would be benefit in future research using qualitative methodology to further explore the meaning and nuances of black humour perceived by mental health professionals.

## Conclusion

Many mental health professionals respond to suffering by using black humour. Black humour is perceived to be beneficial in mental health settings when used mindfully, sensitively and in context. Further study with patients and relatives may shed light on how widely the perception of benefit is shared.

## Data Availability

The authors report direct access to the study data, which have been stored in accordance with New Zealand Ethics Committee (HDECS) guidelines.
